# Ethyl acetate fraction of oregano seed protects non‐alcoholic fatty liver in high‐fat diet‐induced obese mice through modulation of *Srebp‐1c*

**DOI:** 10.1002/fsn3.3939

**Published:** 2024-01-16

**Authors:** Hyun‐Jong Lee, Ji‐Yun Bae, Kye Won Park, Mi‐Ja Kim

**Affiliations:** ^1^ Department of Food and Nutrition, College of Health Science Kangwon National University Samcheok Republic of Korea; ^2^ Department of Food Science and Biotechnology Sungkyunkwan University Suwon Republic of Korea

**Keywords:** lipid metabolism, non‐alcoholic fatty liver disease, obesity, oregano, spice

## Abstract

Oregano (*Origanum vulgare*) seed is used as spices and is known to have anti‐inflammatory, antibacterial, and antioxidant effects. The anti‐fatty liver effects of oregano seed ethyl acetate (OSEA) were evaluated in high‐fat diet (HFD)‐induced obese mice. OSEA was orally administered with HFD for 10 weeks. The body weight, aspartate aminotransferase, alanine aminotransferase, cholesterol, triglyceride, and low‐density lipoprotein levels in the HFD with 100 mg/kg of OSEA significantly decreased by approximately 1.21‐, 1.44‐, 2.12‐, 1.12‐, 1.05, and 1.59 times, respectively, while high‐density lipoprotein levels increased by approximately 1.05 times compared to those in the HFD group (*p* < .05). In addition, the distribution of liver fat in the HFD with 100 mg/kg OSEA (OSEA 100) group decreased significantly (*p* < .05). Therefore, OSEA supplementation can ameliorate fatty liver disease and reduce the accumulation of triglycerides in adipose tissue. The expression of genes involved in liver fat accumulation, such as sterol regulatory element‐binding protein‐1c (*Srebp‐1c*), fatty acid synthase (*Fas*), stearoyl‐CoA desaturase‐1 (*Scd1*), and acetyl‐CoA carboxylase 1 (*Acc1*), significantly decreased in OSEA 100 by approximately 2.6‐, 1.74‐, 1.89‐, and 1.56‐times, respectively (*p* < .05). Therefore, OSEA may modify obesity and liver fat accumulation by regulating the expression of genes involved in lipid metabolism.

## Graphical abstract

- The anti‐fatty liver effects of oregano seed ethyl acetate (OSEA) were evaluated in high‐fat diet‐induced obese mice.
- OSEA supplementation can reduce fatty liver disease and the accumulation of triglycerides in adipose tissue.
- OSEA may modify obesity and liver fat accumulation by down‐regulating gene expression in lipid metabolism.

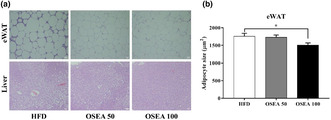

## 1 INTRODUCTION

Non‐alcoholic fatty liver disease (NAFLD) is a term that encompasses diseases in which fatty degeneration of the liver, inflammatory infiltration, and fibrosis occur frequently, along with metabolic diseases such as obesity, insulin resistance, diabetes, and hyperlipidemia (Friedman et al., 2018; Moon et al., 2004). NAFLD is primarily caused by a non‐alcoholic fatty liver, which is formed by excessive triglyceride (TG) accumulation through a high‐fat diet rather than by alcohol absorption (Hong et al., 2020). The development of non‐alcoholic steatohepatitis could be related to NAFLD due to rapid fibrotic progression characterized by hepatic inflammation and hepatocyte expansion (Wong et al., 2018). Recently, the prevalence of NAFLD correlated with obesity increases in Asia as well as in the Western countries.

NAFLD is characterized by highly accumulated TG made of glycerol backbone and fatty acids. In hepatocytes, free fatty acids undergo β‐oxidation to form acyl‐CoA through the activity of acyl‐CoA synthetase (Song et al., 2018). In particular, liver X receptor‐α (LXRα) is a lipid sensor nuclear hormone receptor whose target gene is sterol regulatory element‐binding protein‐1c (SREBP‐1c) and is responsible for the master regulator of hepatic adipogenesis (Kim et al., 2010; Liang et al., 2002). SREBP‐1c regulates the expression of genes involved in lipid metabolism, such as fatty acid synthase (Fasn) and acetyl‐CoA carboxylase (ACC), consequently inducing TG synthesis. Polyphenols suppress the stress generated by reactive oxygen species through their antioxidant activity. These polyphenols are mainly observed in plants and are particularly abundant in herbs. As described above, studies on the treatment and prevention of NAFLD using the antioxidant activity of herbs, which are natural products, have been conducted (Martínez‐Flórez et al., 2005; Salomone et al., 2015; Xiao et al., 2014).

Herbs are used as seasonings to improve the taste of food because of their unique aroma and flavor. Through various studies, herbs possess phytochemicals responsible for the health‐beneficial activities including antibacterial, antioxidant, and anti‐inflammatory activities. Moreover, they are considered as a functional food material with beneficial effects on health (Choi & Rhim, 2008; Dorman & Deans, 2000). Plants that are mainly used as spices include rosemary, oregano, cinnamon, cloves, cumin, and black pepper, which are the main ingredients of curry (Shan et al., 2005). Oregano has a unique taste and is used as a spice in various dishes.

The oregano essential oil component inhibits the growth of food‐poisoning bacteria caused by animal and plant pathogens (Dorman & Deans, 2000). In addition, high antioxidant activity has been shown to inhibit pathogenic fungi, and this antioxidant activity was reported to be due to components of oxidized monoterpene hydrocarbons, such as carvacrol, linalool, and thymol in oregano (Deleanu et al., 2018). Furthermore, the oregano hot water extract showed high antibacterial and antioxidant activities against *Escherichia coli* K99 and *Salmonella typhimurium* (Kang et al., 2017). In our previous study, ethyl acetate fractionated oregano seed demonstrated the better antioxidant activity than other solvent fractions. Among phytochemicals, carvacrol showed the highest content at 19.36% in the oregano seed ethyl acetate fraction (OSEA) (Lee et al., 2021).

Therefore, the aims of this study were to evaluate the oral administration effects of OSEA on NAFLD in high‐fat diet‐induced obese mice and to investigate the expression of genes related to lipogenesis.

## 2 MATERIALS AND METHODS

### 2.1 Preparation of oregano seed ethyl acetate fraction

The ethanol extract and ethyl acetate fraction of oregano seed used in the experiment were prepared in the same manner as in the research method by Lee et al. (2021). Ethanol (80%) per 100 g of oregano seed (Turkey) was added 15 times and mixed for 6 h. The solid was then filtered, concentrated under reduced pressure, and freeze‐dried to prepare an ethanol extract. The freeze‐dried ethanol extract was fractionated in the order of hexane, ethyl acetate, butanol, and water to prepare the ethyl acetate fraction of the oregano seed.

### 2.2 Animals and experimental design

All animal studies were conducted in accordance with the Institutional Animal Care and Use Committee (IACUC) of the College of Biotechnology and Bioengineering at Sungkyunkwan University (protocol number: 2018‐04‐14‐3). Animal studies were performed according to the methods described by Song et al. (2018). Seven‐week‐old male C57BL/6N (Envigo, Indianapolis, IN, USA) mice were purchased and reared in individual cages under a 12‐h light and dark cycle. After a 1‐week adaptation period, each of the nine animals was randomly classified into four groups. Low‐fat diet (LFD; fat 10 kcal%, carbohydrate 70 kcal%, protein 20 kcal%) (Research Diets Inc.), high‐fat diet (HFD; fat 60 kcal%, carbohydrate 20 kcal%, protein 20 kcal%) (Research Diets Inc.), OSEA 50 (HFD + oregano seed ethyl acetate fraction 50 mg/kg/day), and OSEA 100 (HFD + oregano seed ethyl acetate fraction 100 mg/kg/day). Oregano fraction was orally administered for 10 weeks. The LFD and HFD groups were orally administered 5% DMSO in corn oil water (corn oil: water = 1:1), which was used as a vehicle for the oral administration of the oregano fraction (Gad et al., 2006). The body weight and diet of all groups were recorded twice a week.

### 2.3 Glucose tolerance test

The glucose tolerance test (GTT) was done according to the method mentioned (Song et al., 2016). After breeding the mice for 10 weeks in a fasting state for 6 h, basal blood glucose levels were measured (0 min). In fasting conditions, all food was removed, and water supply was stopped before measurement. Subsequently, a glucose solution (2000 mg/kg; Georgiachem Inc., Norcross, GA, USA) was intraperitoneally injected, and glucose in blood was analyzed at 30, 60, 90, 120, and 150 min after injection.

### 2.4 Sacrifice and tissue sampling

The fasting state was maintained for 24 h prior to sacrifice. Blood was collected via cardiac puncture and then immediately refrigerated and centrifuged for 15 min at 3000× *g*. After blood collection, the abdomen was incised to collect subcutaneous fat, epididymal fat, dorsal brown fat, kidneys, spleen, and liver. The kidney and spleen were weighed, and the remaining adipose tissue and liver were stored at −80°C before use.

### 2.5 Blood serum lipid profiling

Serum triglyceride, cholesterol, aspartate aminotransferase (AST), alanine aminotransferase (ALT), high‐density lipoprotein (HDL), and low‐density lipoprotein (LDL) levels were measured using a blood analyzer (Toshiba, Otawara, Japan).

### 2.6 Histological analysis (H&E staining)

The tissues obtained for histological analysis were fixed in 4% paraformaldehyde solution, and paraffin block preparation and staining were performed according to the H&E staining standard protocol (Fischer et al., 2008). The stained adipose and liver tissues were observed using an electron microscope, and the size of the adipose tissue was compared using Image J (LOCI, University of Wisconsin, Madison, WI, USA).

### 2.7 Real‐time PCR analysis

Real‐time PCR was performed according to the method described by Lee et al. (2020). Total RNA extraction from adipose tissue and the liver was homogenized using TRIzol reagent (Invitrogen, Carlsbad, CA, USA). Thereafter, chloroform was added, mixed, allowed to stand for 15 min, and centrifuged at 4°C and 15,000× *g* for 13 min to separate the supernatant. The clear supernatant was transferred to a new e‐tube, mixed with isopropanol, and incubated for 15 min. Subsequently, the pellet thus obtained was washed twice with 75% cold ethanol to extract mRNA. The cDNA synthesis was performed using the qPCR RT Master Mix (TOYOBO, Osaka, Japan). Gene amplification was conducted using real‐time polymerase chain reaction (RT‐PCR, QuantStudio 3, Applied Biosystems, Foster City, CA, USA) with 40 cycles. The expression level of each gene was standardized using beta‐actin, a housekeeping gene, and the average CT value was calculated by repeating each experimental group in triplicate. The primer sequences used to amplify the specific genes are listed in Table 1.

**TABLE 1:** Mice (*mus musculus*) primer sequence.

Gene	Forward primer (5′ → 3′)	Reverse primer (5′ → 3′)
*pparγ*	CCATTCTGGCCCACCAAC	AATGCGAGTGGTCTTCCATCA
*pparα*	ATGCCAGTACTGCCGTTTTC	CCGAATCTTTCAGGTCGTGT
*srebp‐1c*	GGTTTTGAACGACATCGAAGA	CGGGAAGTCACTGTCTTGGT
*fas*	GCTGCTGTTGGAAGTCAGC	AGTGTTCGTTCCTCGGAGTG
*acc1*	GCAGCCCTGGGCACAG	GGGAATACCCGTGGGAGTAGTT
*scd1*	AGAGTAGCTGAGCTTTGGGC	GCATCATTAACACCCCGATAGC
*cd36*	GGCCAAGCTATTGCGACAT	CAGATCCGAACACAGCGTAGA
*nnmt*	TGTGATCTTGAAGGCAACAGA	TTGATTGCACGCCTCAAC
*β‐actin*	CTAGGCACCAGGGTGTGATG	GTCCCAGTTGGTAACAATGCC

Abbreviations: *acc1*, acetyl‐CoA carboxylase 1; *cd36*, cluster of differentiation 36; *fas*, fatty acid synthase; *nnmt*, nicotinamide N‐methyltransferase; *pparα*, peroxisome proliferator‐activated receptor alpha; *pparγ*, peroxisome proliferator‐activated receptor gamma; *scd1*, stearoyl‐CoA desaturase‐1; *srebp‐1c*, sterol regulatory element‐binding protein‐1c.

### 2.8 Western blot analysis

The adipose tissue and liver were lysed using RIPA buffer (Sigma) on ice. Protein was centrifuged at 4°C and 15,000× *g* for 13 min, and the supernatant was separated and extracted. The extracted protein was quantified using a BCA protein assay kit (Thermo Fisher Scientific, Waltham, MA, USA), and the proteins were separated by size using sodium dodecyl sulfate‐polyacrylamide gel electrophoresis (SDS‐PAGE). After SDS‐PAGE, the protein was transferred onto a PVDF membrane, and blocking was performed for 2 h using 5% skim milk in TBST (0.5% v/v Tween‐20 in TBS). The primary antibody (1:1000–2000) was incubated at 4°C overnight, and the secondary antibody (1:2000) was incubated at room temperature for 2 h. The sample was washed at each step six times for 5 min using TBST. Protein band detection was performed on an X‐ray film using an ECL system (Thermo Fisher Scientific) with a membrane.

### 2.9 Statistical analysis

Data are shown as the mean ± SEM using SPSS (SPSS Inc. Chicago, IL, USA). One‐way ANOVA was conducted on the results of body weight and GTT, while other tests were treated with Student's *t*‐test. A *p*‐value <.05 was considered significant.

## 3 RESULTS AND DISCUSSION

### 3.1 Effect of oral administration on OSEA in HFD‐fed obese mice

**FIGURE 1:**
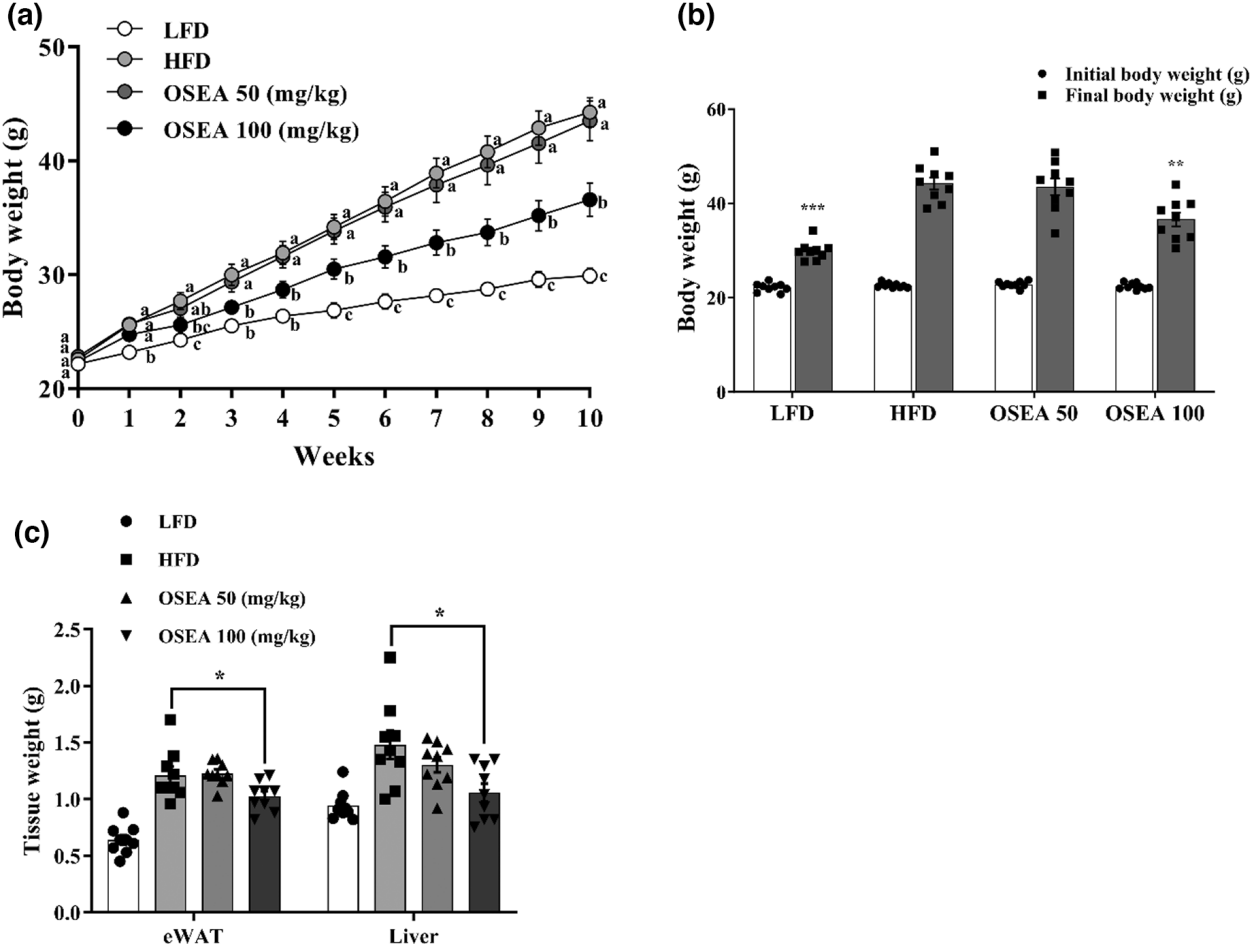
Effect of OSEA on body weight and tissue weight in mice. (a) Body weight in HFD‐fed mice for 10 weeks by OSEA 50 or 100, (b) initial body weight and final body weight in each group, and (c) average weight of epididymal white adipose tissue (eWAT) and liver. Different letters are significant at .05. Symbols of *, **, and *** are significant at .05, .01, and .001, respectively.

The weight changes of each group are shown in Figure 1a. The body weight of the HFD group was significantly increased compared to the LFD group, indicating obesity in HFD group (*p* < .05). The initial body weights of the HFD (22.57 ± 0.17 g), OSEA 50 (22.80 ± 0.22 g), and OSEA 100 (22.40 ± 0.22 g) groups were not significantly different (*p* > .05). However, the body weight of OSEA 100 group and the HFD group showed difference from the second week of oral administration (*p* < .05). After 10 weeks, those of OSEA 100 and the HFD group increased approximately by 63 and 96% compared to the initial body weight, respectively, indicating a significant low body weight gain in OSEA 100 group (*p* < .05) (Figure 1b). As a result of observing the morphology of mice showing the average body weight of each group, the distribution of subcutaneous fat and visceral fat further increased when the HFD was administered compared to the group that received LFD. The weight of epididymal fat significantly decreased by 1.17 times in the OSEA 100 group compared to that in the HFD group (*p* < .05) (Figure 1c). For the aspect of the morphology of the liver tissue, the liver of the HFD group was larger than that of the OSEA group, and the formation of a pale yellow fatty liver was observed. In contrast, the liver tissue of the OSEA group showed a deep scarlet color in a dose‐dependent manner, and the liver tissue weight of the OSEA 100 group significantly reduced by approximately 1.4 times compared to that of the HFD group (*p* < .05) (Figure 1c).

The extracts or essential oils from oregano contain phenolic chemicals including carvacrol and rosmarinic acids, which are known to have antioxidant and antibacterial activities (Ozkan et al., 2010; Rodriguez‐Garcia et al., 2016). Among these physiologically active substances, carvacrol, a phenolic monoterpene component, inhibits the release or synthesis of substances that cause inflammation, thereby exhibiting an anti‐ulcer effect through its anti‐inflammatory activity (Silva et al., 2012). Strong effects of improving obesity by reducing body weight and visceral fat and alleviating plasma lipid levels were reported in mice fed a high‐fat diet (Cho et al., 2012). Main metabolic pathway of carvacrol is conjugation of phenolic groups with glucuronic acid and sulfate (Sharifi‐Rad et al., 2018), whereas those of rosmarinic acid are degraded to caffeic acid (Adomako‐Bonsu et al., 2017; Nadeem et al., 2019). Caffeic acid could inhibit the activities of hepatic fatty acid synthase, 3‐hydroxy‐3‐methylglutaryl‐CoA reductase, and acyl‐CoA: cholesterol acyltransferase resulting in inhibitory effects of fat accumulation (Nadeem et al., 2019).

The ethyl acetate fraction of oregano seed had high antioxidant activity through in vitro experiments, and GC–MS analysis revealed that it contained approximately 19.36% carvacrol as the main component along with phenolic compounds (Lee et al., 2021). The oregano seed ethyl acetate fraction inhibited the differentiation of adipocytes into 3 T3‐L1 pre‐adipocytes, thereby reducing the lipid droplet accumulation and expression of genes related to adipocyte differentiation (Lee & Kim, 2022). The anti‐obesity and non‐alcoholic fatty liver improvement effects in the oregano seed ethyl acetate fraction could be due to the high concentration of carvacrol.

C57BL/6 mice with a high‐fat diet are commonly adapted models for obesity and NAFLD (Recena Aydos et al., 2019; Song et al., 2013). After 10 weeks of oral administration of OSEA along with HFD, the weight gain of the OSEA 100 group significantly reduced compared with that of the HFD group. In addition, the weights of visceral fat and liver tissue were significantly reduced, and the liver tissue was similar to that of mice fed LFD. Amount of daily food intake among the HFD and OSEA groups were not significantly different during the entire feeding period (*p* > .05), indicating that oral administration of OSEA did not affect dietary intake. Therefore, the high antioxidant activity of OSEA may decrease the weight of body, adipose tissue, and liver tissue through inhibiting lipid accumulation.

### 3.2 Effects of OSEA on blood in HFD‐fed obese mice

**FIGURE 2:**
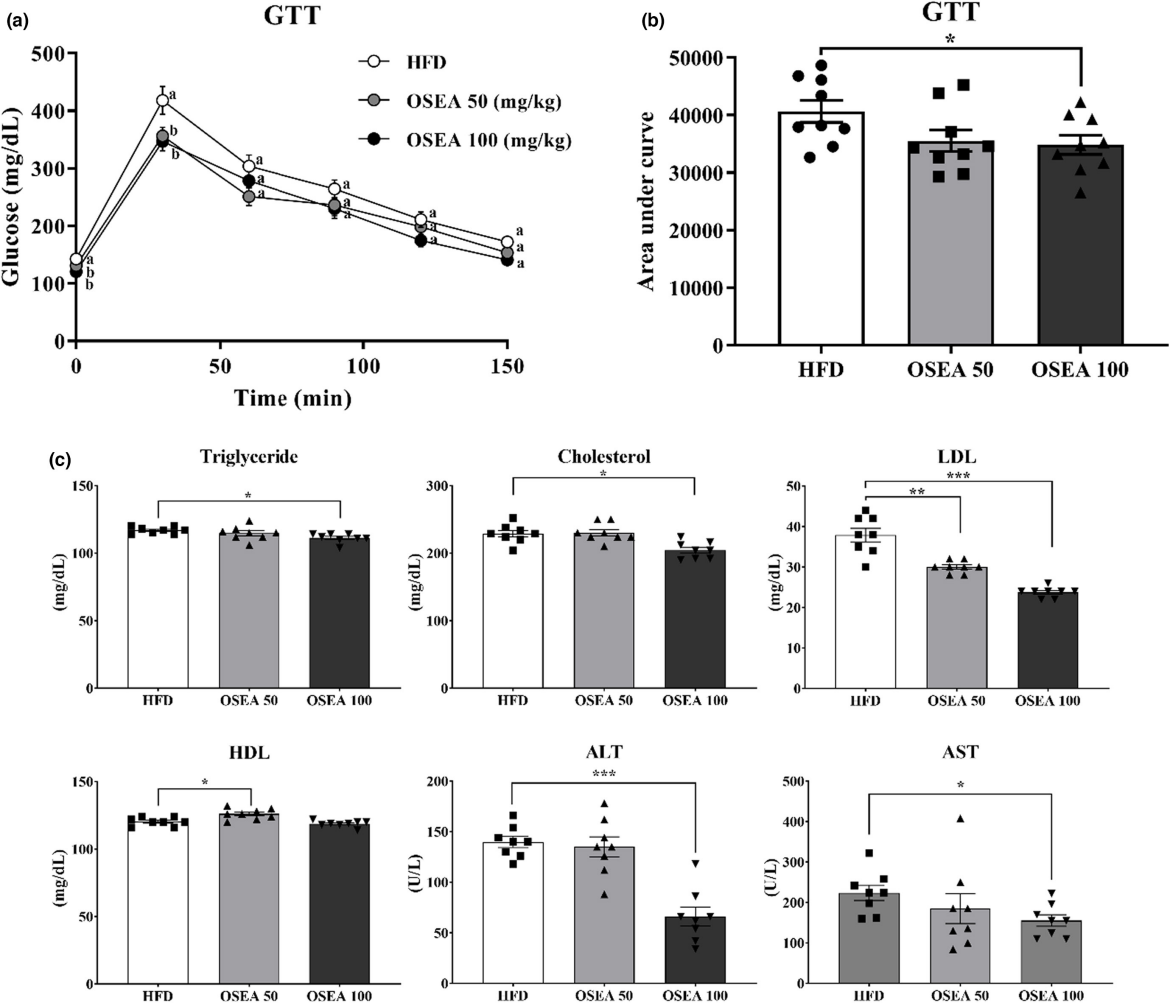
Effect of OSEA on blood biomarkers in mice. (a) Glucose sensitivity, (b) area under curve for glucose sensitivity, and (c) serum biochemical markers including serum concentration of triglyceride, cholesterol, low‐density lipoprotein (LDL), high‐density lipoprotein (HDL), alanine aminotransferase (ALT), and aspartate transaminase (AST). Different letters are significant at .05. Symbols of *, **, and *** are significant at .05, .01, and .001, respectively.

Figure 2a shows the results of the GTT analysis of the HFD, OSEA 50, and OSEA 100 groups. Compared with the HFD group, the OSEA group showed a significant low in initial blood glucose and 30 min after glucose injection (*p* < .05). When the blood glucose values for each time period were expressed as the area under the curve, the difference was significantly reduced by 1.17 times in the OSEA 100 group compared to the HFD group (*p* < .05) (Figure 2b). Oral administration of OSEA can positively affect hepatic glucose metabolism by increasing insulin sensitivity. NAFLD is a disease characterized by hepatocellular steatosis, a state in which more than 5% of the fat accumulates in hepatocytes (Kim et al., 2017). There are various causes of hepatic steatosis, and it is known that it is mainly caused by diet‐induced obesity, insulin resistance, decreased secretion of adiponectin, and decreased free fatty acid in the blood. In particular, insulin resistance and excessive lipid accumulation in the liver are strongly associated (Liu et al., 2010).

The results of the measurement of serum biochemical markers are shown in Figure 2c. Triglycerides and cholesterol significantly decreased in the OSEA 100 group by approximately 1.05 and 1.12 times, respectively, compared to the HFD group (*p* < .05). LDL significantly reduced by 1.26 and 1.59 times in the groups with OSEA 50 and 100, respectively (*p* < .05). HDL significantly increased by approximately 1.05 times in the OSEA 50 group compared to the HFD group (*p* < .05). In the case of ALT and AST, which can confirm whether liver function deteriorated, the OSEA 100 group significantly decreased by approximately 2.12 and 1.44 times compared to the HFD group (*p* < .05). These results indicate that oral administration of OSEA affects lipid metabolism.

It is interesting to note that OSEA leads to a decrease in ALT and AST levels, which could indicate liver function disorder. ALT and AST are enzymes released into the blood when hepatocytes are destroyed, and their detection in the blood indicates liver damage. Oral administration of OSEA can reduce liver damage by protecting liver cells from inflammation or hepatic steatosis. The study of (Botsoglou et al., 2009) observed that AST and ALT levels were significantly reduced when oregano was supplemented in rats induced by oxidative stress with carbon tetrachloride. Thus, oregano dietary supplementation could protect against liver cell damage. According to Rajan et al. (2015), carvacrol treatment in albino rats induced with liver damage using *N*‐nitrosodiethylamine decreased serum AST and ALT levels.

Therefore, oral administration of OSEA can positively affect hepatic glucose metabolism by increasing insulin sensitivity and improving obesity caused by HFD.

### 3.3 Effect of OSEA on epididymal white adipose tissue (eWAT) accumulation and liver steatosis in HFD‐fed obese mice

**FIGURE 3:**
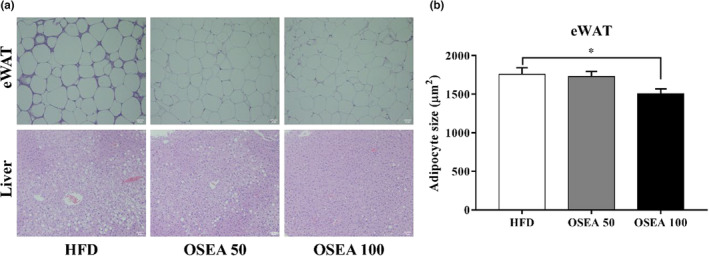
Epididymal adipose and liver tissue in orally administrated mice with OSEA. (a) Representative images of epididymal white adipose tissue (eWAT) and liver through H&E staining of mice and (b) adipocytes size of eWAT. Scale bar = 0.1 μm. Adipocyte size measured used by Image J. Symbol of * is significant at .05.

Morphological analysis through H&E staining in adipose and liver tissues in OSEA is shown in Figure 3a. The size of eWAT adipocytes decreased in the OSEA group compared to that in the HFD group (Figure 3b). In the case of the liver tissue, there was a clear difference between the groups. In the liver tissue of the LFD group, the distribution of lipid droplets was low, and the liver lobules were dark red in color with a generally hexagonal shape. The liver tissue of the HFD group had hepatic steatosis with a significantly higher distribution of adipocytes than the LFD group and was close to pale yellow. However, the distribution of lipid droplets in the liver tissue of the OSEA group gradually decreased in a dose‐dependent manner compared to that in the HFD group. In particular, the OSEA 100 group and the LFD group did not show significant differences in the distribution and color of lipid droplets in the liver tissue. The fat accumulated in the liver mainly consists of triglycerides. Excessive accumulation of triglycerides can lead to toxicity due to fatty acids or fats, resulting in liver damage. Therefore, oral administration of OSEA could suppress fat accumulation in hepatocytes, thereby improving hepatic steatosis.

### 3.4 Effect of OSEA on adipogenic gene expression in eWAT of HFD‐fed obese mice

**FIGURE 4:**
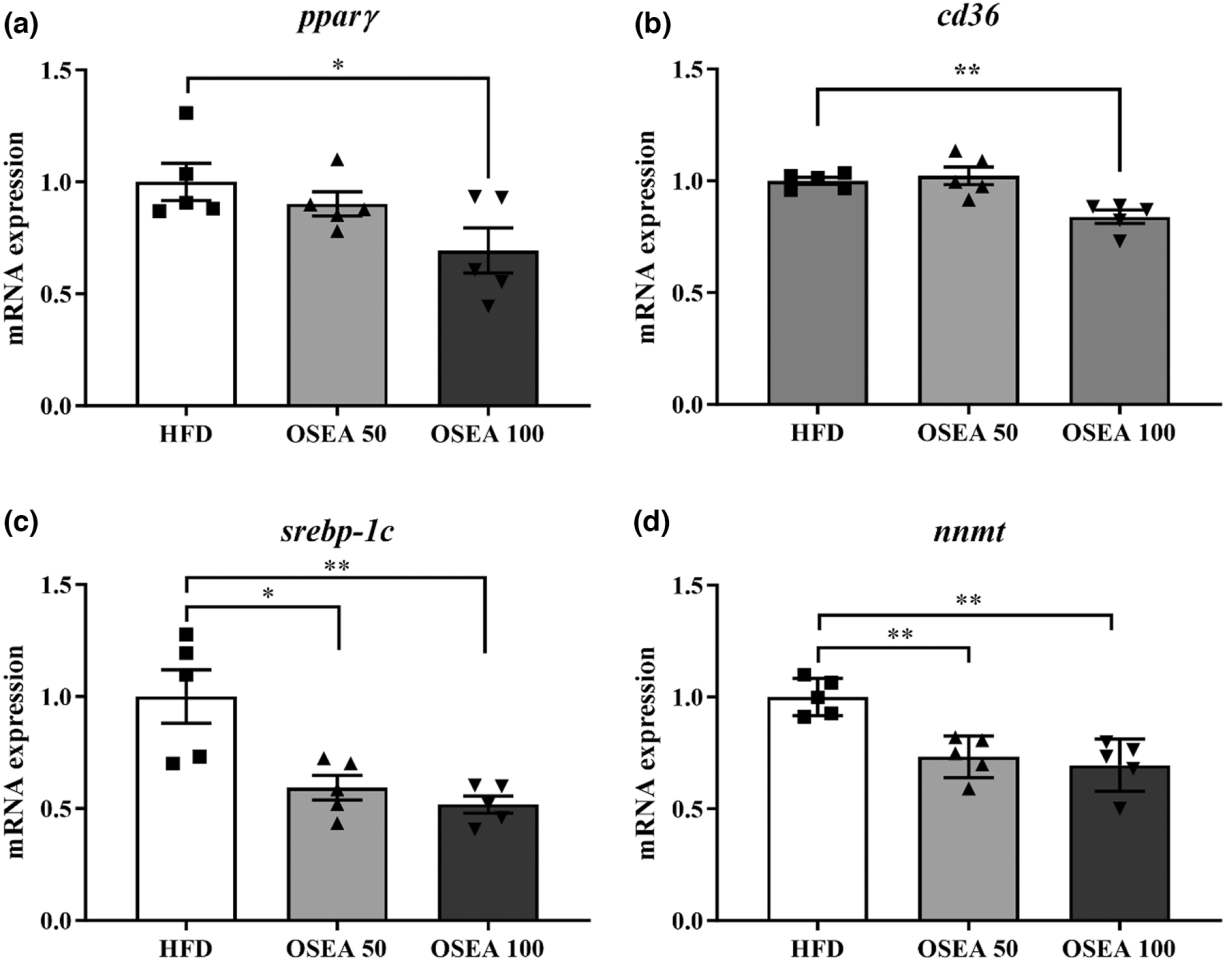
Effect of OSEA on adipose tissue differentiation‐related genes in mice. (a) Peroxisome proliferator‐activated receptor gamma (*pparγ*), (b) cluster of differentiation 36 (*cd36*), (c) Sterol regulatory element‐binding protein‐1c (*srebp‐1c*), and (d) nicotinamide N‐methyltransferase (*nnmt*). Symbols of * and ** are significant at .05 and .01.

The expressions of genes related to adipocyte differentiation in eWAT were analyzed by real‐time PCR (Figure 4). *Pparγ* and *cd36* genes, which are closely related to adipocyte differentiation and adipogenesis, were significantly decreased by 1.44 and 1.19 times, respectively, in the OSEA 100 group (*p* < .05) (Figure 4a,b). *Pparγ* acts as a nuclear receptor that is important in glucose and lipid metabolism. The reduction of *pparγ* inhibits adipogenic differentiation by acting on the initial process of adipocyte differentiation (Han et al., 2002). A decrease in the expression of the reactive gene *cd36* and *pparγ* indicates that the uptake of oxidized lipids is suppressed (Han et al., 2002). Among the *srebps* involved in the synthesis of fatty acids and cholesterol, the expression of *srebp‐1c*, which has an important relationship in the synthesis of fatty acids and triglycerides, was significantly reduced by approximately 1.69 and 1.93 times in the OSEA 50 and 100 groups, respectively (*p* < .05) (Figure 4c). A decrease in the expression of *srebp‐1c* indicates inhibition of fatty acid and triglyceride synthesis (Kohjima et al., 2008). In addition, the expression of *nnmt*, which is closely related to obesity and insulin resistance caused by an increase in adipose tissue, was significantly decreased by approximately 1.36 and 1.44 times in the OSEA 50 and 100 groups, respectively (*p* < .05) (Figure 4d). Decreasing the expression of *nnmt* can improve glucose solubility, thereby ameliorating obesity‐related disease (Ehebauer et al., 2020). OSEA administration may affect the differentiation and the fat accumulation of adipose tissue.

### 3.5 Effect of OSEA on gene expression and protein level associated with hepatic steatosis in liver tissue of HFD‐fed obese mice

**FIGURE 5:**
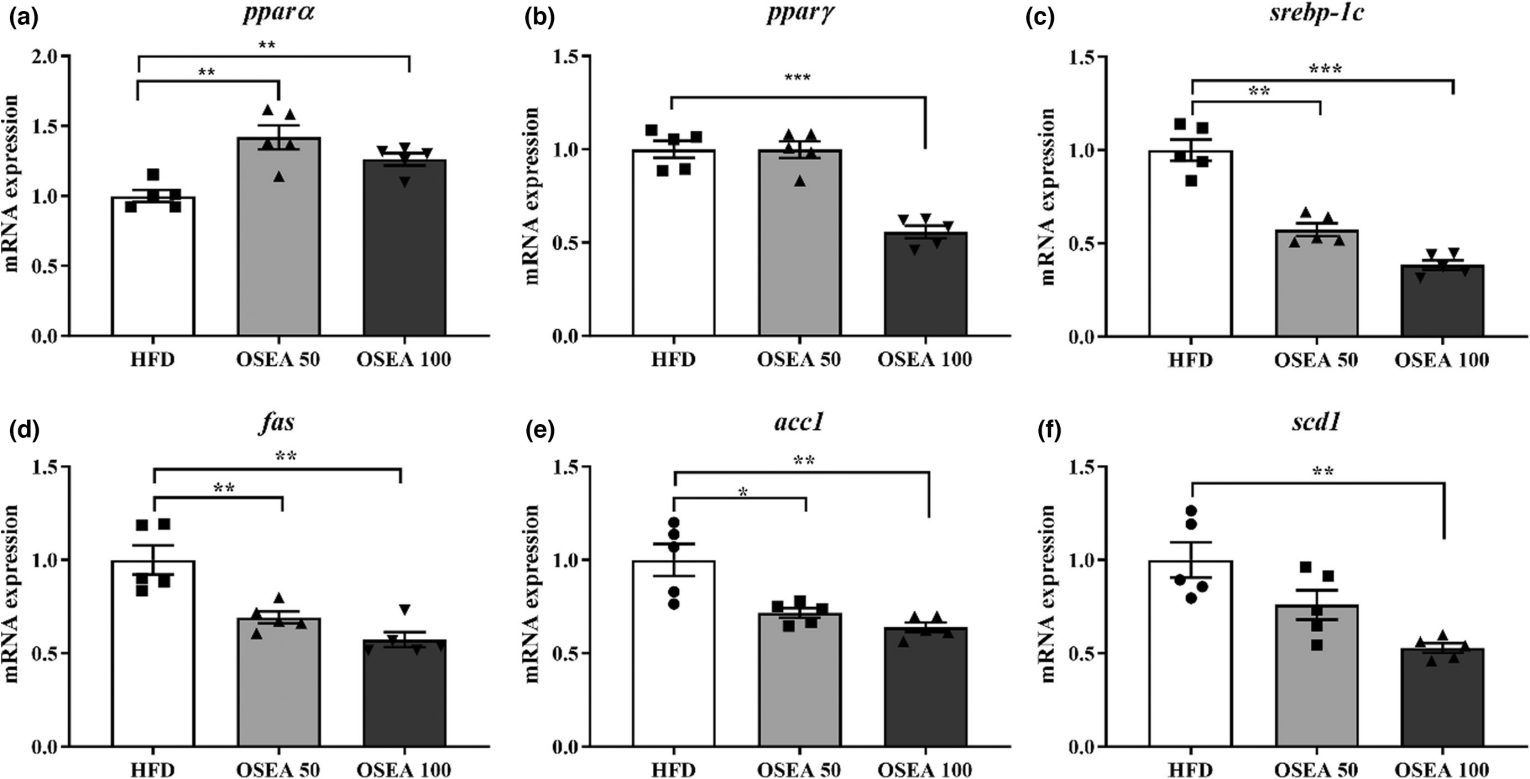
Effect of OSEA on hepatic tissue lipid metabolism‐related genes in mice. (a) Peroxisome proliferator‐activated receptor alpha (*ppar*α), (b) peroxisome proliferator‐activated receptor gamma (*pparγ*), (c) sterol regulatory element‐binding protein‐1c (*srebp‐1c*), (d) fatty acid synthase (*fas*), (e) acetyl‐CoA carboxylase 1 (*acc1*), and (f) stearoyl‐CoA desaturase‐1 (*scd1*). Symbols of *, **, and *** are significant at .05, .01, and .001, respectively.

The mRNA expression is closely related to fatty liver formation and is shown in Figure 5. The expression of *pparα*, an important regulator of fatty acid oxidation and ketogenesis in liver tissue, significantly increased by 1.42 and 1.26 times in the OSEA 50 and 100 groups, respectively (*p* < .05) (Figure 5a). On the other hand, the gene expression of *pparγ*, an adipogenic factor, significantly reduced 1.78 times in the OSEA 100 group (*p* < .05) (Figure 5b). These results indicate that it can inhibit fatty acid synthesis, induce oxidation, and consequently inhibit lipid accumulation in the liver (Kim et al., 2020).

The *srebp‐1c* gene, which is closely related to hepatic steatosis, showed a significant dose‐dependent decrease of 1.75 and 2.63 times in both OSEA 50 and 100 groups, respectively (*p* < .05) (Figure 5c). The expression of *fas* and *acc1* genes related to hepatic lipid metabolism along with *srebp‐1c* significantly decreased by 1.45 and 1.39 times in the OSEA 50 group, respectively, and by 1.75 and 1.56 times in the OSEA 100 group, respectively (*p* < .05) (Figure 5d, e). In the case of *Scd1*, the OSEA 100 group significantly reduced 1.89 times (*p* < .05) (Figure 5f).

**FIGURE 6:**
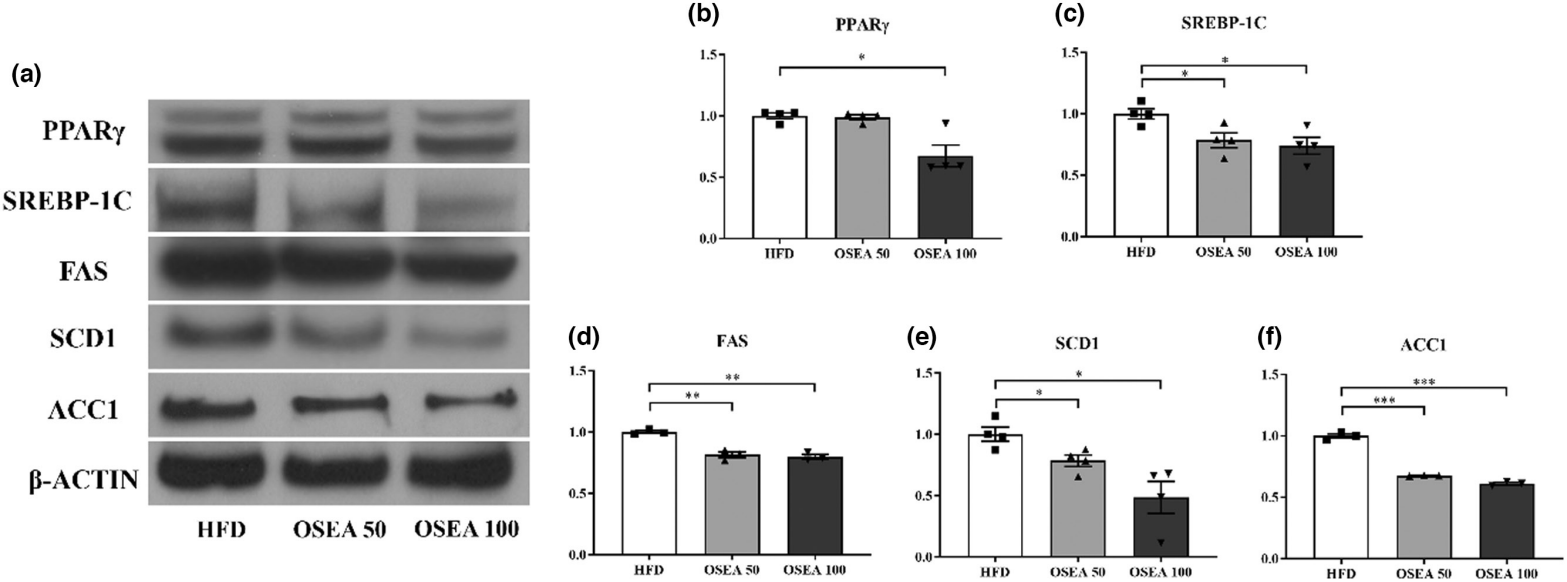
Hepatic tissue lipid metabolism‐related protein expression in OSEA administrated mice. (a) The band of hepatic tissue lipid metabolism‐related proteins, (b) peroxisome proliferator‐activated receptor gamma (PPARγ), (c) sterol regulatory element‐binding protein‐1c (SREBP‐1C), (d) fatty acid synthase (FAS), (e) stearoyl‐CoA desaturase‐1 (SCD1), and (f) acetyl‐CoA carboxylase 1 (ACC1). Symbols of *, **, and *** are significant at .05, .01, and .001, respectively.

The protein levels related to the fat accumulation are shown in Figure 6a. PPARγ expression levels significantly reduced two times in the OSEA 50 group (*p* < .05) (Figure 6b). The expression of SREBP‐1C protein significantly decreased by 1.27 and 1.35 times in the OSEA 50 and OSEA 100 groups, respectively (*p* < .05) (Figure 6c). In the case of FAS, SCD1, and ACC1, which are enzymes related to lipid metabolism, there were no significant differences in FAS protein expression (Figure 6d). However, the protein expressions of SCD1 and ACC1 significantly decreased by 2.04 and 1.64 times, respectively, in the OSEA 100 group (*p* < .05) (Figure 6e, f). *Srebp‐1c* is known to be a major regulator of de novo lipogenesis (DNL) in the liver, and *srebp‐1c* overexpression in the liver induces fatty liver (Song et al., 2018). Citric acid produced during the DNL process is converted to acetyl CoA through ATP‐citrate lyase and further converted to malonyl‐CoA by ACC1. Subsequently, it is converted to palmitic acid by FAS and then undergoes the action of SCD1 to produce complex fatty acids (Jiang et al., 2005; Wakil & Abu‐Elheiga, 2009). As a result of this study, suppression of the expression of genes such as *fas*, *acc1*, and *scd1* along with *srebp‐1c*, a major regulator of DNL, suggests that OSEA may influence regulation of the expression of genes acting on the DNL pathway in the liver.

## 4 CONCLUSION

In this study, anti‐obesity and anti‐hepatic steatosis effects were evaluated by oral administration of an oregano seed ethyl acetate fraction with high antioxidant activity in mice induced by a high‐fat diet. After 10 weeks of oral administration, the OSEA group showed significant weight loss, and the weight of eWAT and liver tissue showed a significant difference. The OSEA group showed significantly decreased plasma triglyceride, cholesterol, LDL, AST, and ALT levels, and significantly increased HDL. Genetic analysis of eWAT tissue revealed that the expression of *pparγ, cd36*, *srebp‐1c*, and *nnmt* related to adipocyte differentiation, significantly decreased in the OSEA group compared to the HFD group. OSEA group decreased the gene expression level of *pparγ*, *srebp‐1c*, *fas*, *acc1*, and *scd1* and protein level related to adipogenic differentiation and hepatic DNL production pathways. OSEA could be used as a natural material for anti‐obesity and fatty liver improvement.

## AUTHOR CONTRIBUTIONS

**Hyun‐Jong Lee:** Formal analysis (equal); writing – original draft (equal). **Ji‐Yun Bae:** Formal analysis (equal). **Kye Won Park:** Supervision (equal); writing – original draft (equal). **Mi‐Ja Kim:** Supervision (equal); writing – original draft (equal).

## CONFLICT OF INTEREST STATEMENT

The authors declare no conflict of interest.

## ETHICAL APPROVAL

All animal experimentations were maintained and carried out with the Institutional Animal Care and Use Committee (IACUC) of the College of Biotechnology and Bioengineering at Sungkyunkwan University (protocol number:2018‐04‐14‐3).

## Data Availability

The data that support the findings of this study are available no request from the corresponding author.
